# Knockdown XIST alleviates LPS‐induced WI‐38 cell apoptosis and inflammation injury via targeting miR‐370‐3p/TLR4 in acute pneumonia

**DOI:** 10.1002/cbf.3392

**Published:** 2019-05-08

**Authors:** Yena Zhang, Yuyin Zhu, Guosheng Gao, Zhiming Zhou

**Affiliations:** ^1^ Department of Pulmonary Medicine, HwaMei Hospital University Of Chinese Academy Of Sciences Ningbo China; ^2^ Department of Laboratory, HwaMei Hospital University Of Chinese Academy Of Sciences Ningbo China

**Keywords:** JAK/STAT and NF‐κB pathways, miR‐370‐3p, pneumonia, XIST

## Abstract

Pneumonia is an inflammatory disease that occurs in the lungs associated with pathogens or other factors. It has been well established that long noncoding RNA X inactivate‐specific transcript (XIST) is involved in several cancers. The present study focused on the effect and detailed mechanism of XIST in lipopolysaccharide (LPS)‐induced injury in pneumonia. Here, XIST was silenced by transfection with XIST‐targeted siRNA, and then, mRNA expression, cell viability, apoptosis, and protein expression were, respectively, assessed by qRT‐PCR, CCK‐8, flow cytometry, and Western blotting. Luciferase reporter, RIP, and RNA pull‐down assays were used to detect the combination of miR‐370‐3p and XIST. Besides, the tested proinflammatory factors were analysed by qRT‐PCR and Western blot, and their productions were quantified by ELISA. The results showed that XIST expression was robustly increased in serum of patients with acute‐stage pneumonia and LPS‐induced WI‐38 human lung fibroblasts cells. Functional analyses demonstrated that knockdown of XIST remarkably alleviated LPS‐induced cell injury through increasing cell viability and inhibiting apoptosis and inflammatory cytokine levels. Mechanistically, XIST functioned as a competitive endogenous RNA (ceRNA) by effectively binding to miR‐370‐3p and then restoring TLR4 expression. More importantly, miR‐370‐3p inhibitor abolished the function of XIST knockdown on cell injury and JAK/STAT and NF‐κB pathways. Taken together, XIST may be involved in progression of cell inflammatory response, and XIST/miR‐370‐3p/TLR4 axis thus may shed light on the development of novel therapeutics to the treatment of acute stage of pneumonia.

**Significance of the study:**

Our study demonstrated that XIST was highly expressed in patients with acute stage of pneumonia. Knockdown of XIST remarkably alleviated LPS‐induced cell injury through increasing cell viability and inhibiting apoptosis and inflammatory cytokine levels through regulating JAK/STAT and NF‐κB pathways.

## 1 INTRODUCTION

Pneumonia is an inflammatory disease, which is characterized by infection of pathogens including bacteria, viruses, and fungi in lower respiratory tracts.^1^, ^2^ Pneumonia represents an important cause of mortality rates worldwide, especially in children and the elderly mainly accompanied with typically clinical symptoms of fever, cough, shortness of breath, chest pain even respiratory failure and heart failures.^3^, ^4^, ^5^ It is well known that pneumonia is correlated with inflammatory stimulation from microbial pathogens (endotoxin etc), which is widely believed to be one of the causes of severe pneumonia.^6^ As a potent endotoxin, lipopolysaccharide (LPS) is the main bioactive component of the cell wall of gram‐negative bacteria and is critical for the inflammatory response.^7^ Thereby, it is imminent to clarify the potential mechanism and develop novel effective strategies about inflammation response in pneumonia for improving the clinical treatment of pneumonia.

Long noncoding RNAs (lncRNAs) have a molecular size of longer than 200 nucleotides and similar to mRNAs in transcription and processing but have no protein‐coding capacity.^8^, ^9^ Currently, increasing evidences have demonstrated that numerous lncRNAs are critical modulators participated in specific physiological and pathological processes via transcriptional or post‐transcriptional regulatory mechanisms.^10^ Fast‐growing number of studies have disclosed that dysregulations of lncRNAs are correlated with various diseases including cancers,^11^ neurodegeneration diseases,^12^ cardiovascular diseases,^13^ and inflammatory diseases, as well as lung cancer^14^ and pulmonary fibrosis.^15^ The lncRNA X inactivate‐specific transcript (XIST), which is located on the X chromosome,^16^, ^17^ is enriched in multiple cancers and regulates tumorigenesis and development as a potential oncogene gene through competing RNA mechanisms.^18^, ^19^, ^20^, ^21^, ^22^ Of note, XIST is proved to regulate the inflammatory response in neuropathic pain and peripheral nerve injury.^23^, ^24^, ^25^, ^26^ Hence, it was hypothesized that XIST might function to modify the inflammation response in pneumonia.

Mounting evidence reveals that lncRNAs harbouring miRNA response elements (MREs) could regulate cancer‐related gene expression via competitively target miRNAs through binding to MREs.^27^ miRNA is a class of short noncoding RNAs with about 20 to 22 nucleotides and negatively regulates protein expression by targeting its 3′ untranslated region (UTR) of mRNA.^28^ Among them, miR‐370‐3p exerts anti‐inflammatory effects in varied diseases, including atherosclerosis, hepatic ischemia‐reperfusion injury, and cardiovascular disease.^29^, ^30^, ^31^ Based on the above research, miR‐370‐3p is capable of reducing the levels of inflammatory cytokines including IL‐6 and IL‐1β through mediating inflammation‐related signalling pathways. Additionally, there are predicted possible binding sites for miR‐370‐3p and XIST. Above all, it is worth exploring whether XIST is able to interact with miR‐370‐3p thus playing a regulatory role in pneumonia.

In this study, XIST was detected highly expressed in serum of acute‐stage pneumonia. To verify our hypothesis that whether XIST could regulate pneumonia development, pneumonia and lung injury cell model was constructed by LPS induction in WI‐38 cells as described in some previous studies.^32^, ^33^ Furthermore, knockdown of XIST inhibited the LPS‐induced cell viability, apoptosis, and inflammatory damage. Moreover, mechanism study shows that XIST acts as an endogenous sponge to increase Toll‐like receptor 4 (TLR4) expression via direct binding miR‐370‐3p and inhibiting its expression. Our results also identified that JAK/STAT and NF‐κB signalling pathway was blocking by XIST knockdown. Here, we provide the first evidence for the regulatory impact of XIST through modulating miR‐370‐3p/TLR4 axis in pneumonia, thus providing new idea for pneumonia diagnosis and treatment.

## 2 MATERIALS AND METHODS

### 2.1 Patients and sample collection

A total of 30 patients diagnosed as acute‐stage pneumonia (mean age, 25.5 ± 3.2 years; 24 males and 6 females) and 30 healthy individuals (mean age, 26.0 ± 2.4 years; 23 males and 7 females) from the Ningbo No. 2 Hospital between June 2016 and July 2017 recruited in this study. Exclude patients with other complications or patients who had received anti‐inflammatory treatment. Three millilitres of fasted peripheral venous blood was collected, centrifuged, and stored at −80°C. Each patient signed a written informed consent in advance, and the study was approved by the ethics committee of Ningbo No. 2 Hospital.

#### Cell culture and LPS treatment

Normal human fibroblast WI‐38 cell line (ATCC; Manassas, VA, USA) were incubated in Dulbecco's modified Eagle's medium (DMEM; Invitrogen, Carlsbad, CA) supplemented with 10% (*v*/*v*) fetal bovine serum and 1% penicillin/streptomycin (Invitrogen) in a humidified atmosphere 5% CO_2_ at 37°C. Cells (2 × 10^5^) were plated in 6‐well plates and incubated 24 hours. LPS in different concentrations (5, 10, and 20 μg/mL) was added in medium for 12 hours to establish injury model.^32^, ^34^, ^35^

### 2.2 Cell transfections

The full length of XIST was amplified from human cDNA by PCR (forward primer 5′‐CCAAGCTTTGCACACGGCCTATCTCATC‐3′ and reverse primer 5′‐CCGCTCGAGTGAAAAGAGGTGGGGCATCC‐3′) and inserted into pcDNA3.1 vector (Invitrogen, USA). The siRNA of XIST, miR‐370‐3p mimics, inhibitor, and the respective negative controls were synthesized and purchased from Applied Biological Materials (GenePharma, Shanghai, China). The siRNA sequence of XIST was si‐XIST#1, 5′‐GCCCUUCUCUUCGAACUGUTT‐3′, si‐XIST#2, 5′‐GUAUCCUAUUUGCACGCUATT‐3′. Cell transfection was executed using Lipofectamine 2000 reagent (Invitrogen), according to the manufacturer's instructions.

### 2.3 RNA extraction and qRT‐PCR

Total RNA was isolated using Trizol reagent (Invitrogen) and next synthesized to first‐strand cDNA using M‐MLV Reverse Transcriptase kit (Invitrogen). qRT‐PCR was performed using SYBR PrimeScript RT‐PCR Kits (Takara, Japan). miR‐370‐3p and other mRNA expression levels were normalized to U6 and β‐actin by 2^−ΔΔCt^ method, respectively. Primers are detailed in Table SS1.

### 2.4 Cell viability assay

The cell viability was assessed using the CCK‐8 proliferation detection kit (Dojindo, Tokyo, Japan). Briefly, a total of approximately 5 × 10^3^ cells were seeded in 96‐well plates; after LPS stimulation, 20 μL CCK‐8 solution was added to each well and cultivated for 1 hour. The absorbance of the reaction system was measured spectrophotometrically at 450 nm.

### 2.5 Detection of cell apoptosis

Flow cytometry analysis for apoptosis was performed with Annexin V FITC‐propidium iodide (PI) staining assay (Invitrogen, CA, USA) based on the manufacturer's instruction.

### 2.6 Enzyme‐linked immunosorbent assay

WI‐38 cell culture supernatant in 24‐well plates was collected, and the productions of inflammatory cytokines were measured by enzyme‐linked immunosorbent assay (ELISA) kits (R&D Systems, Minneapolis, MN, USA) as instructed and quantified by normalization to protein concentrations.

### 2.7 Western blotting

Cells were lysed with lysis buffer containing protease inhibitors (50 mM Tris‐HCl pH 8, 50 mM NaCl, 0.5% NP‐40). Equal amounts of protein were resolved by sodium dodecyl sulfate‐polyacrylamide gel electrophoresis (SDS‐PAGE) gels and transferred to PVDF membranes. After blocking with 5% nonfat milk, the membranes were then immunoblotted with primary antibodies against TLR4, Bcl‐2, Bax, cleaved caspase‐3, cleaved caspase‐9, IL‐6, IL‐1β, TNF‐α, JAK2, p‐JAK2, STAT3, p‐STAT3, p65, p‐p65, IkBα, p‐IkBα, and β‐actin (Abcam, USA) at a dilution of 1:1000 overnight at 4°C. Following washing, the membranes were exposed to HRP‐conjugated goat anti‐rabbit (1:5000, Santa Cruz); secondary antibodies and signals were detected with ECL detection system.

### 2.8 Luciferase reporter assay

WI‐38 cells were cultured into 24‐well plates and transfected with pmirGLO dual‐luciferase vector (XIST WT, XIST MUT, TLR4 WT, and TLR4 MUT) together with miR‐370‐3p mimics or negative control (NC mimics). After 24 hours, luciferase activity was measured by the dual‐luciferase reporter assay system (Promega, Madison, WI, USA).

### 2.9 RNA immunoprecipitation assay

RNA immunoprecipitation (RIP) experiment was conducted using Magna RIP RNA‐binding protein immunoprecipitation kit (Millipore, Billerica, MA, USA) and the Ago2 antibody (Abcam, Cambridge, MA, USA) according to the manufacturer's instruction. The expression of XIST was analysed by qRT‐PCR. Normal mouse IgG (Abcam, USA) and anti‐SNRNP70 (Abcam, USA) were, respectively, used as negative control and positive control.

### 2.10 RNA pull‐down assay

Purified RNAs were, respectively, labelled and transcribed with Biotin RNA Labeling Mix (Roche, Switzerland) and T7 RNA polymerase (Ambion Life) in vitro and purified with an RNeasy Plus Mini Kit (Qiagen, Valencia, CA, USA) and DNase I (Qiagen). Purified RNAs, positive control, negative control, and biotinylated RNAs were mixed and incubated with AGS cell lysates. Then, magnetic beads were added to each binding reaction and incubated at room temperature. RNA complexes bound to these beads were eluted and extracted for qRT‐PCR analysis.

### 2.11 RNA‐FISH

Cy3‐labelled XIST and DAPI‐labelled U6 probes were designed and synthesized by RiboBio (China). A fluorescent in situ hybridization kit was used to perform RNA‐FISH assay according to the manufacturer's protocol (Thermo Fisher).

### 2.12 Statistical analysis

All quantitative data were presented as mean ± SD and analysed using SPSS 20.0 software (SPSS Inc., Chicago, IL, USA). The comparison significances were, respectively, evaluated by a two‐sided Student's *t* test for two groups and one‐way ANOVA test for three or more groups. All experiments were repeated at least three times. *P* < .05 was considered as statistically significant.

## 3 RESULTS

### 3.1 lncRNA XIST upregulated in serum of pneumonia patients

**Figure 1:**
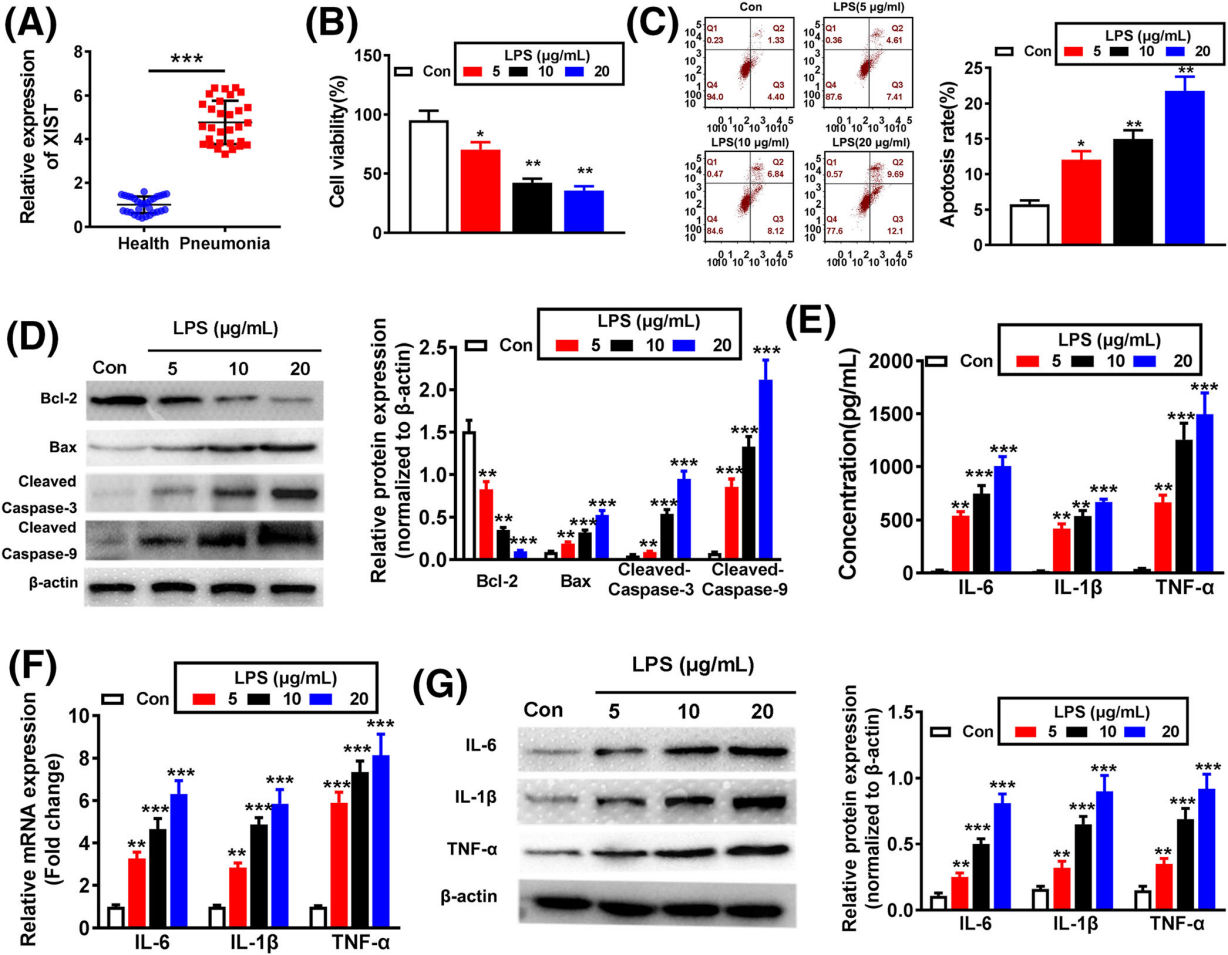
X inactivate‐specific transcript (XIST) expression in pneumonia patients and lipopolysaccharides (LPS) induces inflammatory damage in WI‐38 cells. A, XIST expression in serum of acute‐stage pneumonia patients and healthy controls was detected by qRT‐PCR. B, The effect of different concentrations of LPS (5, 10, and 20 μg/mL) on cell viability was estimated by CCK‐8 assay. The effect of LPS on cell apoptosis was evaluated by (C) flow cytometry and (D) Western blotting. The expression levels of IL‐6, IL‐1β, and TNF‐α were evaluated by (E) ELISA, (F) qRT‐PCR, and (G) Western blotting after treatment with LPS. *P < .05, **P < .01, and ***P < .001

The results showed that XIST was significantly increased in acute‐stage pneumonia than that in healthy control (Figure 1A). Hence, we speculated that implying XIST might play a potential role in regulating acute pneumonia development.

### 3.2 LPS stimulation induced cell apoptosis and inflammation injuries in WI‐38 cells

To examine the functions of LPS on WI‐38 cells, different concentrations of LPS (5, 10, and 20 μg/mL) were used to induce cell injury. CCK‐8 assay demonstrated that cell viability was obviously reduced as with increasing LPS concentration (Figure 1B). Furthermore, the cell apoptosis was significantly increased with increasing concentration of LPS (Figure 1C). Meanwhile, LPS induced the decreased protein level of antiapoptotic marker Bcl‐2, while increased the protein levels of proapoptotic markers including Bax, cleaved caspase‐3, and cleaved caspase‐9 (Figure 1D). By performing ELISA assay, LPS evidently increased the secretion of proinflammatory cytokines (IL‐6, IL‐1β, and TNF‐α) (Figure 1E). In agreement, qRT‐PCR and Western blotting assays indicated that the mRNA and protein levels of the above‐mentioned proinflammatory factors were also significantly decreased (Figure 1F,G). Since 10 μg/mL LPS contributed to an obvious reduction in cell viability while significant increase in cellular inflammatory damage, 10 μg/mL was selected for the following experimental conditions. These data suggest that LPS‐induced acute pneumonia WI‐38 cell model is successfully constructed.

### 3.3 XIST was upregulated in LPS‐induced WI‐38 cells

XIST was reported to promote cancer development and could cause inflammation response in chronic constriction injury, neuropathic pain, and chronic pain.^23^, ^24^, ^25^, ^36^ To profile the expression of XIST in LPS‐injured WI‐38 cells, qRT‐RCR analysis was evaluated. The results demonstrated that XIST expression is significantly evaluated at different degrees in WI‐38 cells with LPS treatments at various levels.

### 3.4 Inhibition of XIST alleviated cell apoptosis and inflammation injuries in LPS‐induced WI‐38 cells

**Figure 2:**
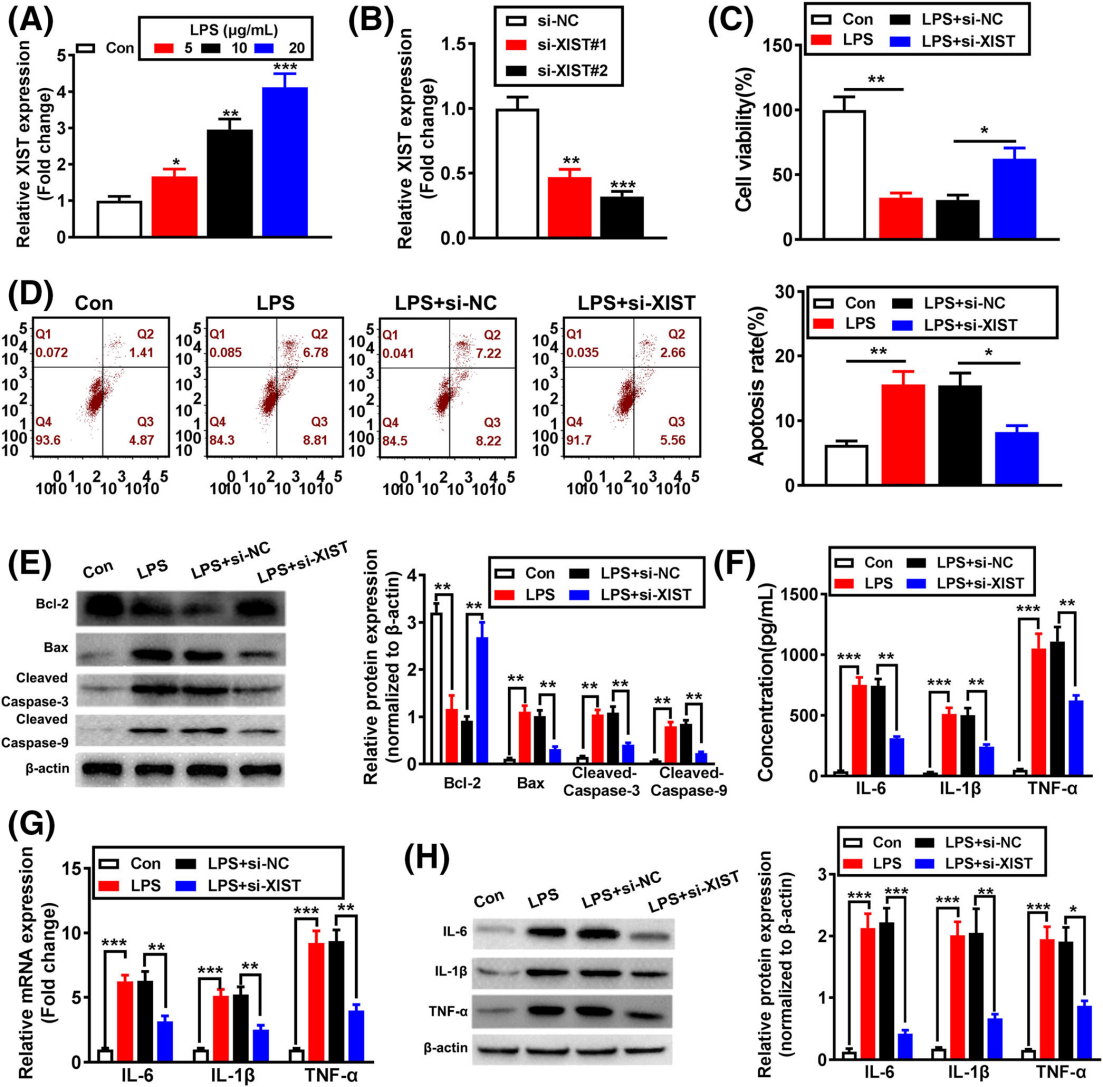
Inhibition of X inactivate‐specific transcript (XIST) alleviated cell apoptosis and inflammation injuries in lipopolysaccharide (LPS)‐induced WI‐38 cells. A, XIST expression was detected by qRT‐PCR. B, The transfected efficiency of si‐XIST was analysed by qRT‐PCR. C, The effect of XIST knockdown on cell viability was analysed by CCK‐8 assay. The effect of XIST knockdown on cell apoptosis was evaluated by (D) flow cytometry and (E) Western blotting. The expression levels of IL‐6, IL‐1β, and TNF‐α were analysed by (F) ELISA, (G) qRT‐PCR, and (H) Western blotting. *P < .05, **P < .01, and ***P < .001

To examine the possible functions of XIST in LPS‐induced inflammatory injuries, WI‐38 cells were transfected with siRNA targeting XIST. qRT‐PCR assay was applied to verify the transfection efficiency. More specifically, the data demonstrated that XIST expression was dramatically inhibited by si‐XIST#1 and si‐XIST#2 transfection compared with si‐NC group, and si‐XIST#2 was selected for subsequent experiments because of better knockdown effect (Figure 2B). As a result, XIST knockdown significantly enhanced cell viability and the protein level of Bcl‐2, while decreased apoptotic cell rates, and the protein of Bax, cleaved caspase‐3, and cleaved caspase‐9 (Figure 2C‐E) in response to LPS treatment. Meanwhile, knockdown of XIST also reduced the production of IL‐6, IL‐1β, and TNF‐α in LPS‐induced WI‐38 cells (Figure 2F‐H). Collectively, above findings indicate that XIST can increase inflammatory injuries of LPS‐induced WI‐38 cells.

### 3.5 XIST suppressed miR‐370‐3p expression by functioning as a sponge in LPS‐induced WI‐38 cells

**Figure 3:**
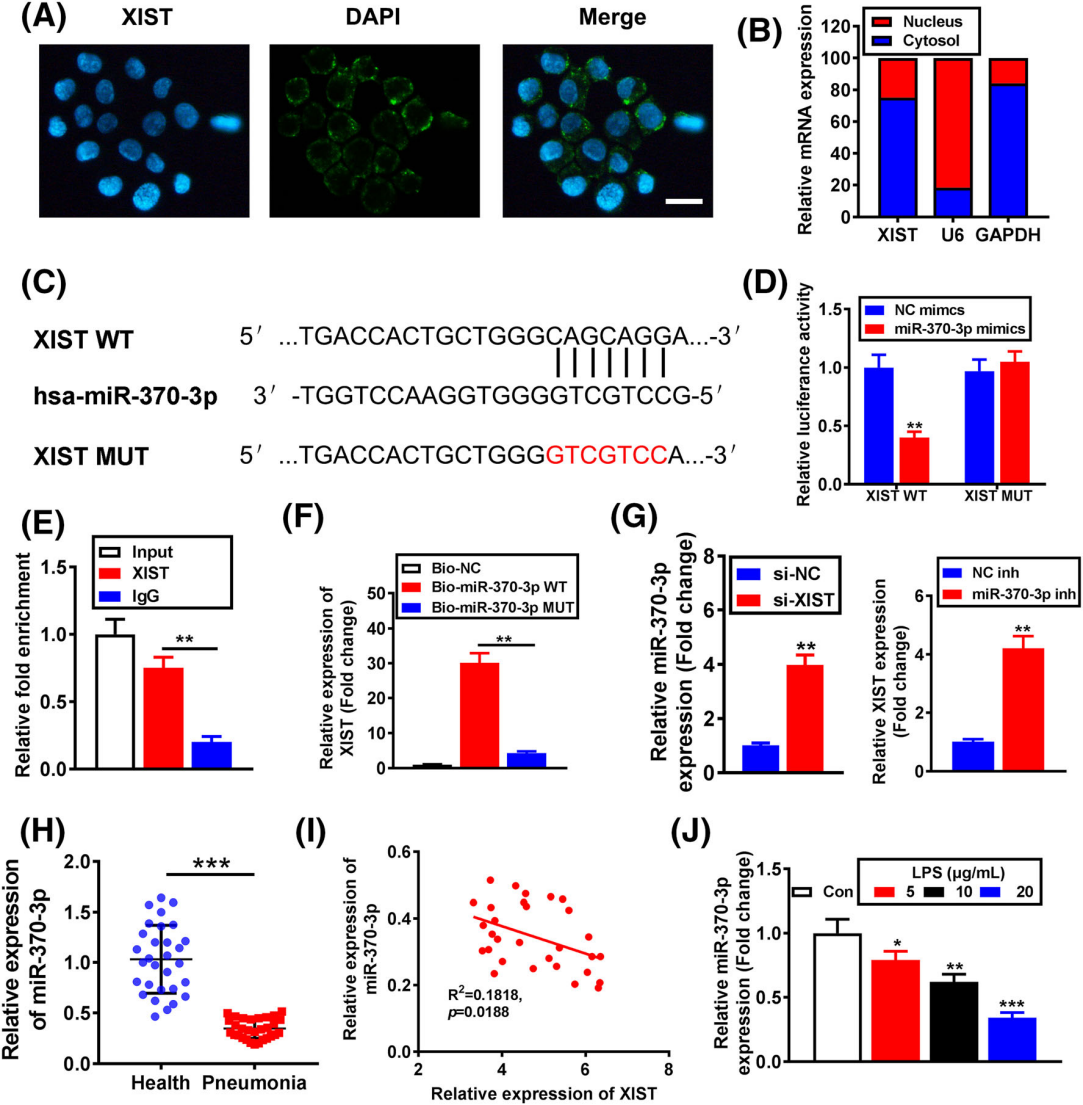
X inactivate‐specific transcript (XIST) suppressed miR‐370‐3p expression by functioning as a sponge in lipopolysaccharide (LPS)‐induced WI‐38 cells. A, Localization of XIST by RNA‐FISH in WI‐38 cells. Nuclei is stained red (DAPI), and XIST is stained blue (scale bar = 50 μM). B, XIST expression in nucleus and cytoplasm was analysed by qRT‐PCR. C, Putative miR‐370‐3p binding sequence and mutation sequence of XIST mRNA were as shown. D, Luciferase reporter assays were used to prove XIST can target miR‐370‐3p. E, Association of miR‐370‐3p and XIST with AGO2 was performed by immunoblotting assays. RNA levels were presented as fold enrichment in Ago2 relative to IgG immunoprecipitates. F, qRT‐PCR was used to detect XIST expression in the sample pulled down by biotinylated miR‐370‐3p WT and miR‐370‐3p MUT probe. G, miR‐370‐3p and XIST expression levels were analysed by qRT‐PCR. H, miR‐370‐3p expression in serum of acute stage pneumonia patients and healthy controls was analysed by qRT‐PCR. I, The correlation analysis between XIST expression and miR‐370‐3p expression in acute‐stage pneumonia patients (n = 30) was performed by Spearman's rank correlation analysis. J, miR‐370‐3p expression was detected by qRT‐PCR. *P < .05, **P < .01, and ***P < .001

The subcellular localization of lncRNA is closely associated with its biological effects and potential molecular roles; thus, RNA‐FISH assay was performed to detect the subcellular distribution of XIST. The results showed that most of punctate patterns were abundant in the cytoplasm, while minority in the nucleus (Figure 3A), which was also verified by nucleocytoplasmic separation experiment (Figure 3B). Aiming to elucidate the exact mechanisms underlying the function of XIST, we searched its potential targets using starBase database (http://starbase.sysu.edu.cn/starbase2/index.php); a putative interaction between XIST and miR‐370‐3p was found, and binding sites of wild type (XIST‐WT) and mutant type (XIST‐MUT) were shown (Figure 3C). Luciferase reporter assay demonstrated that XIST‐WT and miR‐370‐3p mimic cotransfection significantly reduced luciferase activity, while XIST‐MUT and miR‐370‐3p mimic cotransfection failed to affect luciferase activity in WI‐38 cells (Figure 3D). The interaction between miR‐370‐3p and XIST was further validated in RIP assay, and the results indicated that XIST exerted more significant enrichment in the Ago2‐containing miRNA ribonucleoprotein complexes (miRNPs) than the control IgG immunoprecipitates (Figure 3E). Meantime, biotin‐labelled pull‐down assay demonstrated that XIST was efficiently pulled down by bio‐miR‐370‐3p (Figure 3F), but not bio‐miR‐370‐3p MUT. Consistently, qRT‐PCR analysis indicated that XIST inhibition upregulated miR‐370‐3p expression, while miR‐370‐3p inhibitor considerably enhanced XIST expression in WI‐38 cells (Figure 3G). Moreover, the serum miR‐370‐3p expression in acute‐stage pneumonia patients was significantly decreased compared with healthy control (Figure 3H), and the level of XIST and miR‐370‐3p exhibited a dramatically negative correlation confirmed by Spearman's correlation analysis (Figure 3I). Furthermore, miR‐370‐3p expression was signally reduced with LPS induction in WI‐38 cells in a concentration‐dependent manner (Figure 3J). On the basis of the above findings, XIST might exert an effect on miR‐370‐3p deregulation through functioning as a sponge.

### 3.6 TLR4 was a target gene of miR‐370‐3p

**Figure 4:**
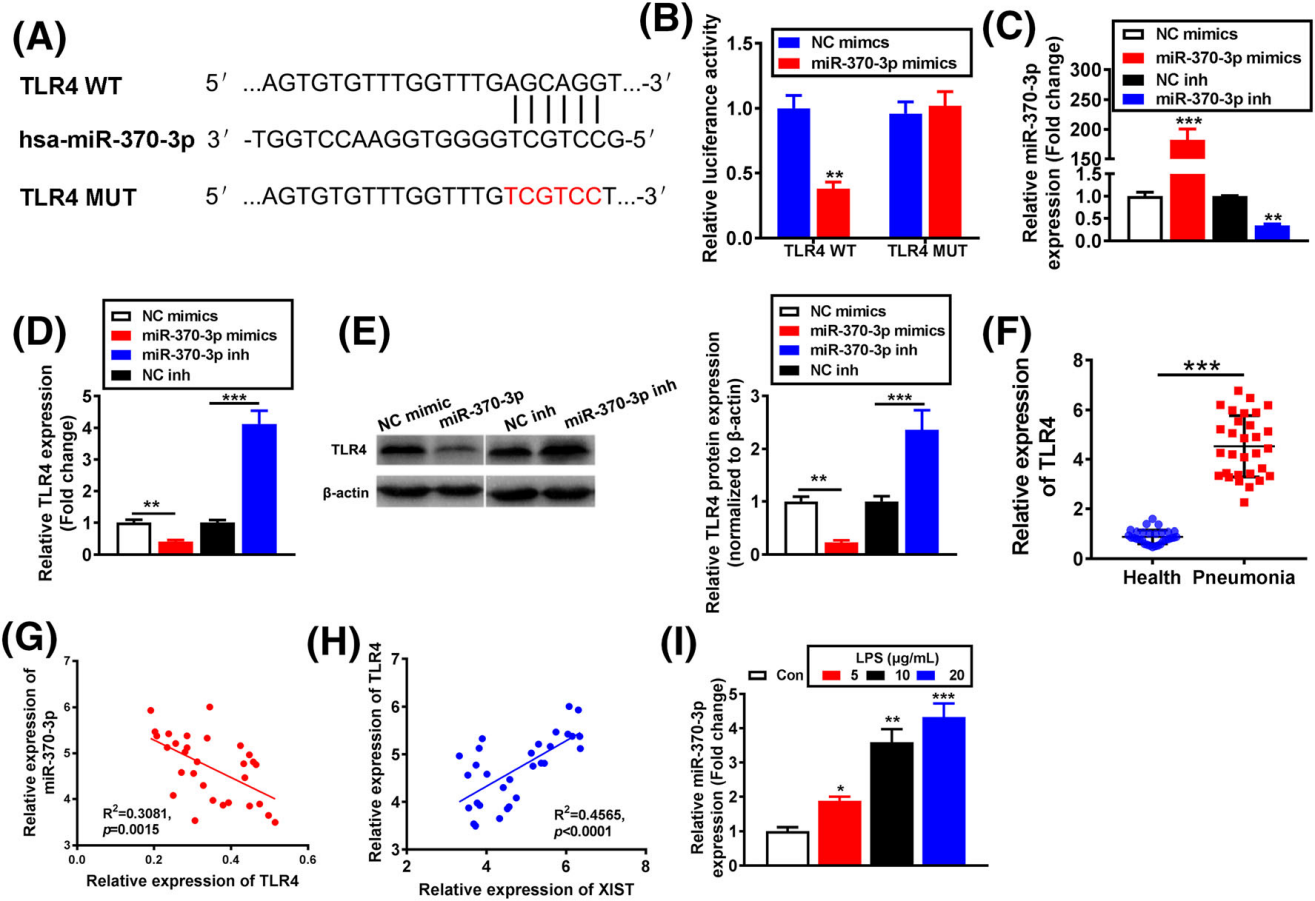
TLR4 was a target gene of miR‐370‐3p. A, Putative miR‐370‐3p binding sequence and mutation sequence of TLR4 mRNA were as shown. B, Luciferase reporter assays were used to prove that miR‐370‐3p can target TLR4. C, The transfection efficiency by miR‐370‐3p mimics, and inhibitor was analysed by qRT‐PCR. TLR4 expression was analysed by (D) qRT‐PCR and (E) Western blotting. F, CCL5 expression in serum of acute stage pneumonia patients and healthy controls was analysed by qRT‐PCR. The correlation analysis (G) between TLR4 and miR‐370‐3p expression and the correlation analysis (H) between TLR4 and X inactivate‐specific transcript (XIST) expression in acute stage pneumonia patients (n = 30) were performed by Spearman's rank correlation analysis. I, TLR4 expression was analysed by qRT‐PCR. *P < .05, **P < .01, and ***P < .001

Recent study reported that miR‐370‐3p inhibited vascular inflammation and oxidative stress by targeting TLR4 in ox‐LDL‐incubated THP‐1 cells,^29^ but the potential effect of miR‐370‐3p on TLR4 expression in WI‐38 cells is still unclear. Bioinformatics tool TargetScan database (http://www.targetscan.org/) predicted the putative binding sites between miR‐370‐3p and TLR4. Mechanically, the binding sites and modified sequence in the TLR4 3' UTR are shown in Figure 4A. Dual‐luciferase reporter assay suggested that the luciferase activity of cells with TLR4‐WT transfection was significantly decreased by miR‐370‐3p mimics, while there was no alteration in TLR4‐MUT‐transfected group (Figure 4B). To further confirm this result, miR‐370‐3p mimics or inhibitor was transfected in WI‐38 cells, and transfection efficiency was evaluated by qRT‐PCR (Figure 4C). Further, qRT‐PCR proved that the mRNA level of TLR4 in WI‐38 cells was significantly reduced by miR‐370‐3p mimics, while increased by miR‐370‐3p inhibitor (Figure 4D). Meanwhile, Western blotting also confirmed that the TLR4 protein level were reduced or enhanced by miR‐370‐3p mimics or inhibitor, respectively (Figure 4E). The above data demonstrated that TLR4 were a direct target of miR‐370‐3p. Moreover, the serum TLR4 expression in acute‐stage pneumonia patients was obviously higher than healthy control (Figure 4F). Next, Spearman's correlation analysis proved that XIST expression was positively while miR‐370‐3p expression was inversely correlated with TLR4 expression in acute‐stage pneumonia patients (Figure 4G,H). In addition, TLR4 mRNA expression was significantly increased with LPS induction in a concentration‐dependent manner (Figure 4I). Altogether, the results above proved that TLR4 was a target gene of miR‐370‐3p and was positively modulated by XIST.

### 3.7 Downregulation of XIST inhibits LPS‐induced apoptosis and inflammatory damage via miR‐370‐3p in LPS‐injured WI‐38 cells

**Figure 5:**
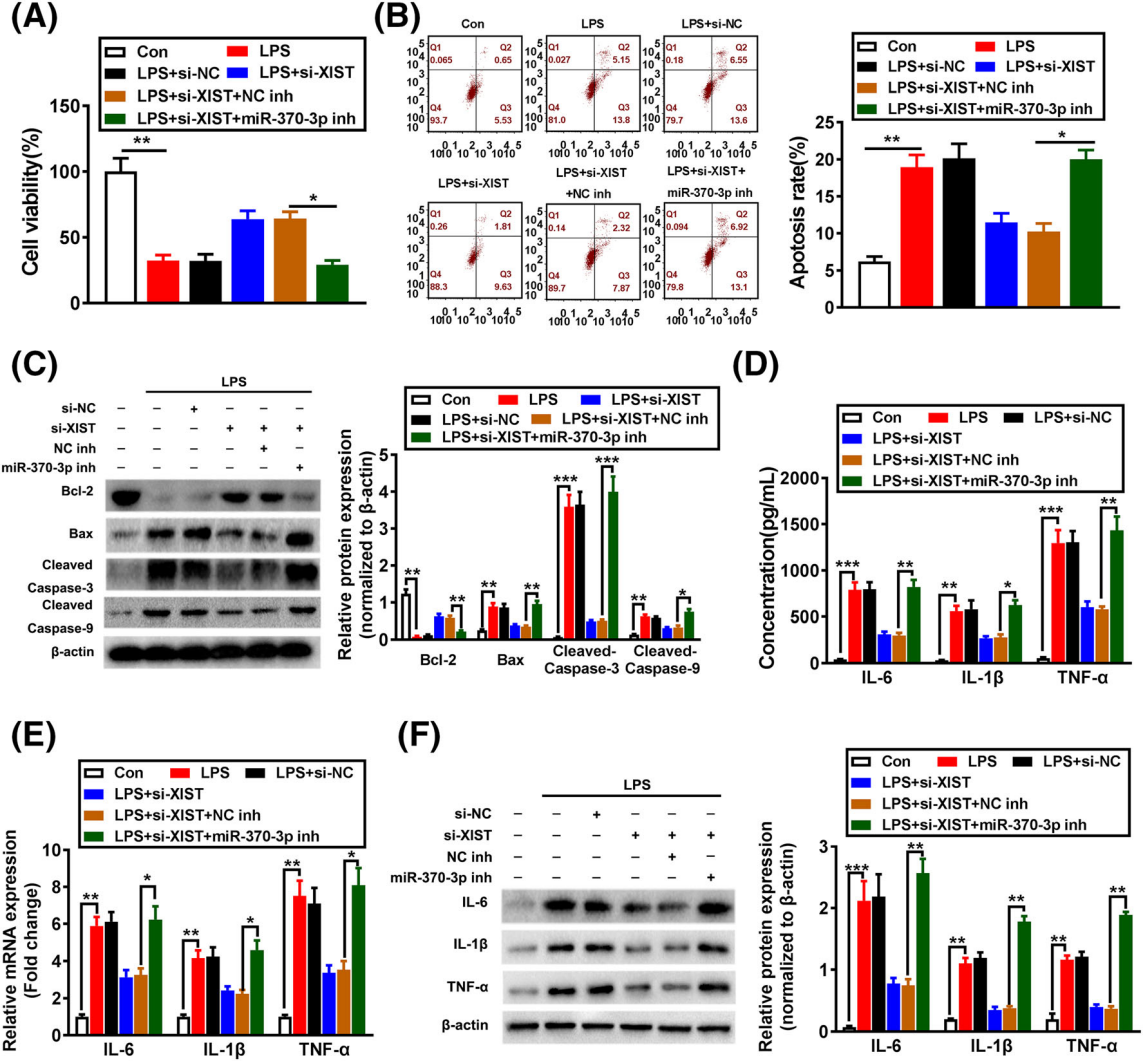
Downregulation of X inactivate‐specific transcript (XIST) inhibits LPS‐induced apoptosis and inflammatory damage via miR‐370‐3p in LPS‐injured WI‐38 cells. A, Cell viability was analysed in si‐NC, si‐XIST transfected or si‐XIST + NC inhibitor, or si‐XIST + miR‐370‐3p inhibitor cotransfected WI‐38 cells after LPS treatment by CCK‐8 assay. Cell apoptosis in si‐NC, si‐XIST transfected or si‐XIST + NC inhibitor, or si‐XIST + miR‐370‐3p inhibitor cotransfected WI‐38 cells after LPS treatment was evaluated by (B) flow cytometry and (C) Western blotting. IL‐6, IL‐1β, and TNF‐α expression levels were detected by (D) ELISA, (E) qRT‐PCR, and (F) Western blotting in si‐NC, si‐XIST transfected or si‐XIST + NC inhibitor, or si‐XIST + miR‐370‐3p inhibitor cotransfected WI‐38 cells after LPS treatment. *P < .05, **P < .01, and ***P < .001

To explore the regulatory relationship between XIST and miR‐370‐3p, XIST was downregulated by its siRNA, and miR‐370‐3p expression was further knocked down by its inhibitor in WI‐38 cells. As expected, si‐XIST improved cell viability and reduced apoptosis, whereas the effect of XIST silencing was largely abrogated by the concomitant inhibition of miR‐370‐3p (Figure 5A,B). Congruously, the function of si‐XIST on increasing Bcl‐2 and decreasing the Bax, cleaved caspase‐3, and cleaved caspase‐9 protein levels was abolished by cotransfection with miR‐370‐3p inhibitor (Figure 5C). Furthermore, decreased inflammatory factors (IL‐6, IL‐1β, and TNF‐α) by XIST inhibition was also reversed by miR‐370‐3p inhibition (Figure 5D,F). These results imply that XIST aggravates LPS‐induced WI‐38 cell injuries through targeting miR‐370‐3p.

### 3.8 XIST regulated JAK/STAT and NF‐κB pathways in LPS‐induced WI‐38 cells

**Figure 6:**
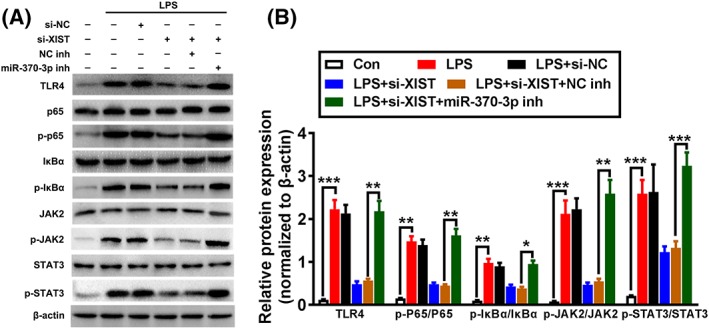
X inactivate‐specific transcript (XIST) regulated JAK/STAT and NF‐κB pathways in LPS‐induced WI‐38 cells. A, Representative western blotting results for TLR4, p65, p‐p65, IkBα, p‐IkBα, JAK2, p‐JAK2, STAT3, and p‐STAT3 protein expression from si‐NC, si‐XIST transfected or si‐XIST + NC inhibitor, or si‐XIST + miR‐370‐3p inhibitor cotransfected WI‐38 cells after LPS treatment. B, Quantitative analysis of proteins was obtained by using β‐actin as a control. *P < .05, **P < .01, and ***P < .001

The effect of XIST on the JAK/STAT and NF‐κB pathway in LPS‐induced WI‐38 cells was further analysed. As shown in Figure 6A,B, LPS treatment markedly led to the increased expression levels of TLR4, p‐p65, p‐IkBα, p‐JAK2, and p‐STAT3, while this increase effect was obviously inhibited by si‐XIST. Nevertheless, the effects of XIST knockdown on the expressions of these proteins were dramatically reversed by cotransfection with si‐XIST and miR‐370‐3p inhibitor. These data indicated that knockdown of XIST inhibits LPS‐induced activation of JAK/STAT and NF‐kB pathways through regulating miR‐370‐3p expression.

## 4 DISCUSSION

Pneumonia is one of the common respiratory tract inflammatory diseases especially among children and the elderly. A large body of evidence indicated that cytokine accumulation and inflammation function a substantial role in pneumonia development. The release of inflammatory factors, such as TNF‐α, could promote cell apoptosis, microvascular dysfunction, and tissue necrosis.^37^ The cumulative message of several recent studies has revealed that lncRNAs are abnormally involved in inflammation response and serve as effective therapeutic targets for pneumonia.^32^, ^35^ We put forward biological functions and mechanisms of XIST in pneumonia and firstly found that XIST knockdown lead to the inhibition of cell apoptosis and inflammation injuries in LPS‐induced WI‐38 cells by sponging miR‐370‐3p. Furthermore, TLR4 was identified as a target of miR‐370‐3p, and function of XIST knockdown on LPS‐injured WI‐38 cells was weakened by miR‐370‐3p inhibition. In addition, XIST knockdown blocked LPS‐induced JAK/STAT and NF‐kB pathways.

XIST has been demonstrated to play oncogenic function in multiple tumours. For example, XIST regulates colorectal cancer development and metastasis by the competition of miR‐200b‐3p to regulate ZEB1 expression.^38^ Meanwhile, XIST regulates the growth, invasion, and migration of bladder cancer by interacting with miR‐124 to target androgen receptor.^39^ Not only in cancer, current literature researches prove that XIST also can affect inflammation response. Sun et al showed that XIST inhibition could reduce inflammatory pain through inhibiting Nav1.7 by acting as a sponge for miR‐146a, suggesting a promising strategy for fighting inflammatory pain.^23^ Moreover, XIST knockdown inhibited neuroinflammation by suppressing TNF‐α, COX‐2, and IL‐6 expression in CCI rats.^24^, ^25^ However, there are few literature studies on the function and mechanism of XIST in pneumonia.

And recent research has shown that ceRNA hypothesis is proposed to explain a new regulatory mechanism of lncRNA through sponging miRNAs in many diseases.^40^ Our studies suggest that XIST negatively regulates miR‐370‐3p to promote apoptosis and inflammation in LPS‐induced WI‐38 cells. Tian et al revealed that miR‐370‐3p can reduce inflammation factor level including IL‐6 and IL‐1β and inhibit ROS accumulation via targeting TLR4 in THP‐1 cells,^29^ indicating the potential relation between miR‐370‐3p and pneumonia. Although the role of miR‐370‐3p in pulmonary injury caused by pneumonia has not investigated, based on our study, we speculate that downregulation of miR‐370‐3p may aggravate pulmonary injury.

Additionally, TLR4 was identified as a functional target of miR‐370‐3p. Toll‐like receptor is involved in nonspecific immunity, and TLR4 is the first identified mammalian TLR and serves a vital function in inflammatory responses.^41^ Notably, previous studies have suggested that TLR4 signalling can promote injury, inflammation, and pulmonary fibrosis in acute or chronic lung injury mice model.^42^ TLR4 recruits signal transducers and initiates signal cascades through ligand binding, which can active NF‐κB pathway and promote the production of inflammatory factors, such as IL‐6 and TNF‐α.^43^ Meanwhile, the JAK/STAT signalling pathway has been involved in pathological inflammatory response.^44^ More importantly, JAK/STAT and NF‐κB signalling has been implicated in pneumonia. For example, miR‐1247 could aggravate acute pneumonia through activating JNK and NF‐κB pathways, and CRNDE can upregulate FOXM1 that further activates NF‐κB and JAK/STAT pathways in LPS‐injured WI‐38 cells.^35^, ^45^ Because of the key function of JAK/STAT and NF‐κB in pneumonia, our data prove that XIST knockdown contributes to inhibition of JAK/STAT and NF‐kB pathways through sponging miR‐370‐3p during pneumonia progression.

In conclusion, this study firstly indicated that XIST was increased in serum of patients with acute‐stage pneumonia and LPS‐injured WI‐38 cells; downregulation of XIST attenuated LPS‐induced apoptosis and inflammation in WI‐38 cells via modulating miR‐370‐3p/TLR4 axis. This study may supply novel clues for understanding the role of XIST in pneumonia progression and offer strategies for therapeutic approach to acute stage of pneumonia.

## AVAILABILITY OF DATA AND MATERIALS

The datasets used in the present study are available from the corresponding author on reasonable request.

## ETHICS APPROVAL AND CONSENT TO PARTICIPATE

The present study was approved by the Ethical Committee of Ningbo No. 2 Hospital (Ningbo, China).

## COMPETING INTERESTS

The authors declare that there is no conflict of interest regarding the publication of this paper.

## Supporting information

Table S1. Primer sequences used for amplification
