# Supplementary material for: Knockdown XIST alleviates LPS‐induced WI‐38 cell apoptosis and inflammation injury via targeting miR‐370‐3p/TLR4 in acute pneumonia
Source: Cell Biochem Funct. 2019 May 8;37(5):348–58. doi: 10.1002/cbf.3392 (PMC6618287; doi:10.1002/cbf.3392)
Supplement: Supplementary file 1 — Table S1. Primer sequences used for amplification [file CBF-37-348-s001.doc]

**Supplementary Table ​1. Primer sequences used for amplification**

| Name | Sequecences |
| --- | --- |
| XIST | F: 5′-CGGGTCTCTTCAAGGACATTTAGCC-3′  R: 5′-GCACCAATACAGAGGAATGGAG-3′ |
| miR-370-3p | F:5′-TGTAACCAGAGAGCGGGATGT-3′  R: 5′-TTTTGGCATAACTAAGGCCGAA-3′ |
| TLR4 | F:5′-AGAAACTGCTCGGTCAGACG -3′  R: 5′-GGGCTAAACTCTGGATGGGG -3′ |
| IL-6 | F: 5′-GAAATCGTGGAAATGAG-3′  R: 5′-TAGGTTTGCCGAGTAGA-3′ |
| IL-1β | F: 5′-GCCCTAAACAGATGAAGTGCTC-3′  R: 5′-GAACCAGCATCTTCCTCAG-3′ |
| TNF-α | F: 5′-GCCAATGGCATGGATCTCAAAG-3′  R: 5′-CAGAGCAATGACTCCAAAGT-3′ |
| U6 | F:5′-TGCGGGTGCTCGCTTCGGCAGC-3′  R: 5′-CCAGTGCAGGGTCCGAGGT-3′ |
| β-actin | F:5′-TGGAATCCTGTGGCATCCATGAAAC-3′  R:5′-ACGCAGCTCAGTAACAGTCCG-3′ |
